# Supplementary figures and images for: Clinical characteristics and prognosis of basaloid squamous cell carcinoma of the lung: a population-based analysis
Source: PeerJ. 2019 Apr 30;7:e6724. doi: 10.7717/peerj.6724 (PMC6499056; doi:10.7717/peerj.6724)

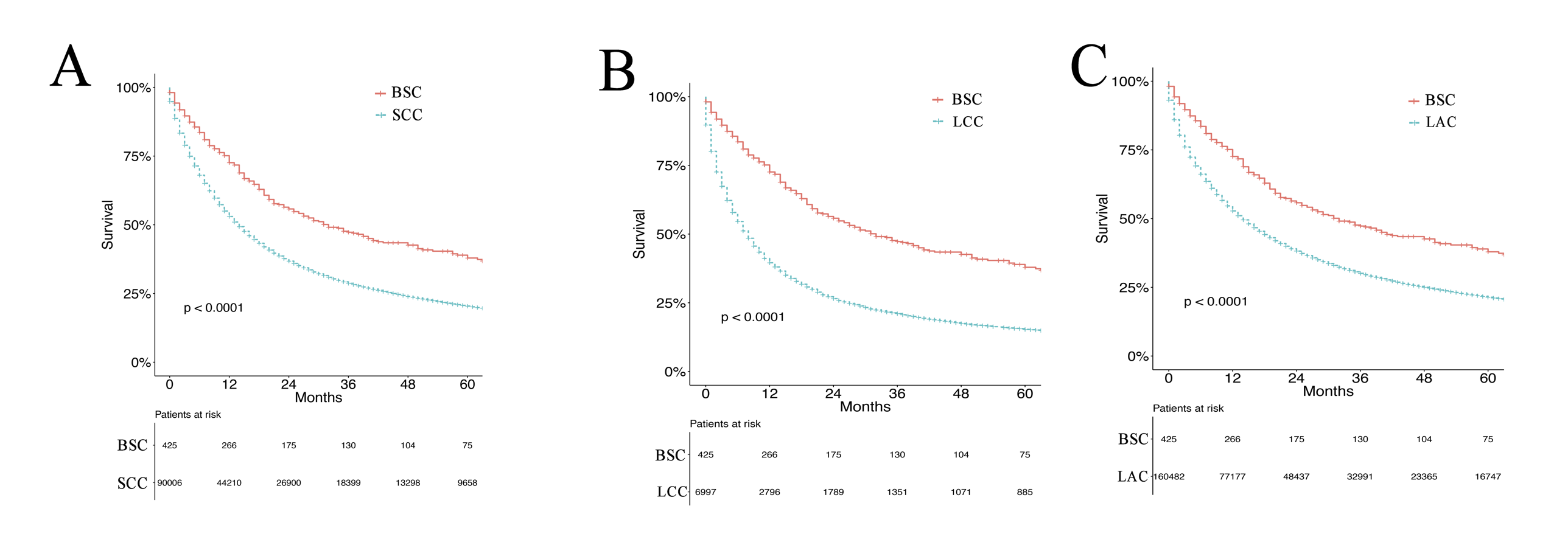

Supplement: Figure S1 — (A) The survival curve of basaloid squamous cell carcinoma of the lung (BSC) and lung squamous cell carcinoma (SCC). (B) The survival curve of basaloid squamous cell carcinoma of the lung (BSC) and large cell carcinoma (LCC). (C) The survival curve of basaloid squamous cell carcinoma of the lung (BSC) and lung adenocarcinoma (LAC). [file peerj-07-6724-s001.png]

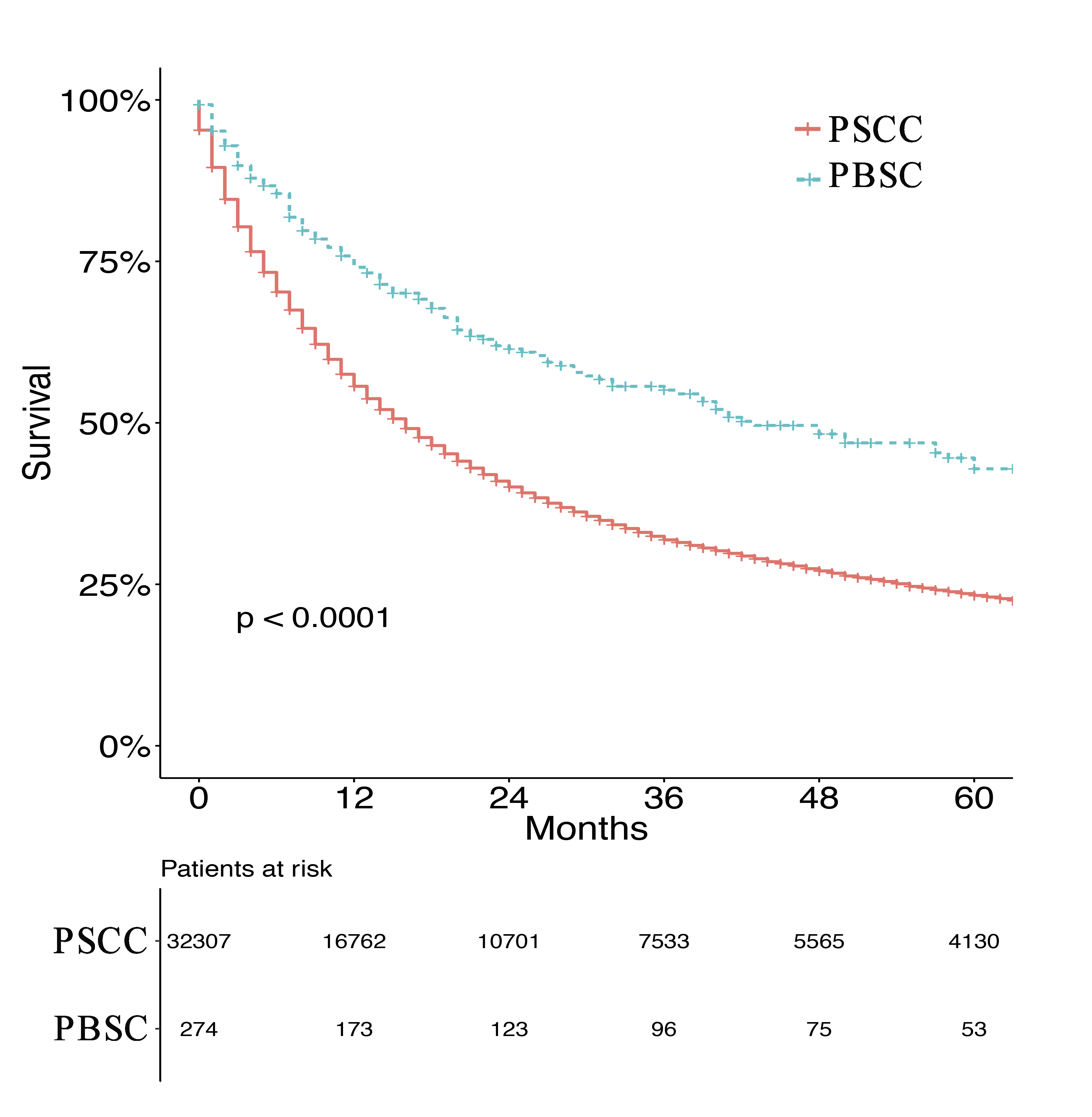

Supplement: Figure S2 — The survival curve of poor differentiated basaloid squamous cell carcinoma of the lung (PBSC) and poor differentiated lung squamous cell carcinoma (PSCC). [file peerj-07-6724-s002.png]
